# Establishment of a Daqu Grade Classification Model Based on Computer Vision and Machine Learning

**DOI:** 10.3390/foods14040668

**Published:** 2025-02-16

**Authors:** Mengke Zhao, Chaoyue Han, Tinghui Xue, Chao Ren, Xiao Nie, Xu Jing, Haiyong Hao, Qifang Liu, Liyan Jia

**Affiliations:** 1 College of Food Science and Engineering, Shanxi Agricultural University, Taigu, Jinzhong 030801, China; z18339058292@163.com (M.Z.); 19726869037@163.com (C.H.); xuetinghui569@163.com (T.X.); renchao2333@outlook.com (C.R.); 13852163371@163.com (X.N.); x.jing@vip.163.com (X.J.); 2 Graduate Education Innovation Center on Baijiu Bioengineering in Shanxi Province, Taigu, Jinzhong 030801, China; 3 Industry Technology Innovation Strategic Alliance on Huangjiu in Shanxi Province, Taigu, Jinzhong 030801, China; 4 Shanxi Xinghuacun Fenjiu Distillery Co., Ltd., Fenyang 032200, China; h19628@126.com; 5 College of Information Science and Engineering, Shanxi Agricultural University, Taigu, Jinzhong 030801, China

**Keywords:** light-flavor Daqu, image segmentation, feature factors, machine learning, evaluation model

## Abstract

The grade of Daqu significantly influences the quality of Baijiu. To address the issues of high subjectivity, substantial labor costs, and low detection efficiency in Daqu grade evaluation, this study focused on light-flavor Daqu and proposed a two-layer classification structure model based on computer vision and machine learning. Target images were extracted using three image segmentation methods: threshold segmentation, morphological fusion, and K-means clustering. Feature factors were selected through methods including mean decrease accuracy based on random forest (RF-MDA), recursive feature elimination (RFE), LASSO regression, and ridge regression. The Daqu grade evaluation model was constructed using support vector machine (SVM), logistic regression (LR), random forest (RF), k-nearest neighbor (KNN), and a stacking model. The results indicated the following: (1) In terms of image segmentation performance, the morphological fusion method achieved an accuracy, precision, recall, F1-score, and AUC of 96.67%, 95.00%, 95.00%, 0.95, and 0.96, respectively. (2) For the classification of Daqu-P, Daqu-F, and Daqu-S, RF models performed best, achieving an accuracy, precision, recall, F1-score, and AUC of 96.67%, 97.50%, 97.50%, 0.97, and 0.99, respectively. (3) In distinguishing Daqu-P from Daqu-F, the combination of the RF-MDA method and the stacking model demonstrated the best performance, with an accuracy, precision, recall, F1-score, and AUC of 90.00%, 94.44%, 85.00%, 0.89, and 0.95, respectively. This study provides theoretical and technical support for efficient and objective Daqu grade evaluation.

## 1. Introduction

Daqu is a saccharification starter for Baijiu production [1], playing a crucial role in the brewing process. It has a long and rich history and is one of the core components of Chinese Baijiu brewing culture [2,3,4]. Daqu is produced through fermentation by mixing various grains, including barley, wheat, and peas. It not only provides an abundant source of microorganisms for the liquor body but also endows Baijiu with unique flavors and aromas through the fermentation process [5]. The type of Daqu used in the production of traditional light-flavor Baijiu is low-temperature Daqu, referred to as light-flavor Daqu [6]. Light-flavor Daqu is made mainly from barley and peas through steps such as raw material mixing and grinding, shaping, solid-state fermentation, and aging [7]. Light-flavor Daqu can be divided into Qingcha Qu (QC), Hongxin Qu (HX), and Houhuo Qu (HH) according to different production processes [8]. Often referred to as “the backbone of liquor,” the quality of Daqu significantly affects the quality and style of Baijiu [9,10,11]; premium Daqu can ensure the smooth progress of the Baijiu fermentation process, produce more beneficial metabolites, and give Baijiu a mellow taste, rich aroma, and harmonious flavor.

Due to the complex fermentation process of Daqu and the lack of unified quality evaluation standards, many enterprises rely on experience to grade Daqu. Experienced artisans judge the grade of Daqu by observing its appearance, color, aroma, and cross-sectional features [12]. Daqu is usually divided into three grades: premium (Daqu-P), first-grade (Daqu-F), and second-grade (Daqu-S) [13]. However, manual judgment is subjective. Due to differences in their own experience, taste sensitivity, and understanding of the Daqu quality evaluation criteria, different tasters often give different results when grading Daqu. Manual grading is also inefficient. In large-scale production, a large number of Daqu samples need to be graded. The manual grading process is time-consuming, labor-intensive, and prone to causing sensory fatigue [14,15,16]. Moreover, the lack of precise and quantitative scientific standards makes it difficult to guarantee the accuracy and stability of Daqu grading.

Therefore, the study on how to reduce the subjectivity of Daqu classification, improve the consistency of raw material quality, and ensure the flavor and quality of the final product is of great significance to the development of the entire Baijiu industry.

With the increasing demand for quality control of agricultural products and their processed products, the application of computer vision and machine learning technology in the quality assessment and classification of agricultural products is increasingly widespread [17]. Scholars Weng et al. integrated computer vision and intelligent sensory technology to build a partial least square prediction model for evaluating the texture and color of chicken and judging its freshness and quality, with R2 reaching 0.94 [18]. Yang used a new hybrid view technology to reduce the impact of the cabbage leaf gap on quality and improve the accuracy of evaluation, with R2 reaching 0.92 [19]. Some scholars used k-nearest neighbor (KNN), extreme learning machine, and partial least squares discriminant analysis algorithms to detect pesticide residues in cantaloupe, and the accuracy reached 93.48%, which played an important role in evaluating the quality and safety of the agricultural products [20]. A study utilized a support vector machine (SVM) and a backpropagation neural network to achieve quality classification and realized quality discrimination by extracting a gray co-occurrence matrix of pear shape, surface color, and defect area, with the classification accuracy reaching 83.30% and 91.00%, respectively [21]. In addition, a rice classification model was successfully established by combining fluorescence hyperspectral technology with machine learning technology, and the accuracy of the random forest (RF) model in the test set reached 95.30% [22]. Using CMOS and near-infrared cameras to construct an intelligent detection system based on the convolutional neural network architecture enabled the implementation of quality control for apples and achieved a bruise detection accuracy of over 97.00% [23]. In recent years, stacking models have been gradually applied in the agricultural field. For example, hyperspectral imaging was combined with stacking ensemble learning to detect wheat saccharification force and protein content [24]. Some studies utilized a stacked model that combined multilayer perceptron with machine learning to improve the prediction accuracy of the dry matter yield of cabbage [25]. In summary, computer vision and machine learning technologies provide efficient and reliable support for the quality assessment of agricultural products and their processed products. However, their application in the classification of Daqu grades remains to be explored.

Daqu image research is still in its infancy. Studies have explored a Daqu quality detection system based on computer vision technology. By detecting the size, cross-sectional cracks, and mold contamination, the correlation between visual features and Daqu quality could be established to evaluate its quality [26]. This indicates that computer vision technology has shown certain potential applications in the quality assessment of Daqu. However, the classification of Daqu images still faces challenges. The introduction of computer vision technology and machine learning algorithms into Daqu grade classification can not only provide a scientific, objective, and efficient method but also provide a more systematic solution to improve the quality evaluation and grade evaluation of Daqu.

This study aimed to develop a Daqu grade classification method based on computer vision and machine learning to improve the accuracy and scientific rigor of Daqu grade evaluation. The primary objectives were (1) to investigate the feasibility and advantages of applying computer vision and machine learning techniques to Daqu image classification; (2) to employ multiple image segmentation techniques, including morphological fusion, threshold segmentation, and K-means clustering, to achieve precise segmentation of Daqu images, focus on specific regions, and extract key image features. Significant discriminative feature factors were selected using feature optimization and screening strategies to provide high-quality inputs for the classification model. A two-layer classification framework was applied to systematically compare the performance and applicability of different machine learning algorithms, and an efficient and reliable Daqu image classification model was constructed. This study not only provided scientific evidence and technical support for Daqu grade classification but also offered valuable insights into the application of machine learning techniques in food quality inspection.

## 2. Materials and Methods

### 2.1. Sample Collection

**Figure 1:**
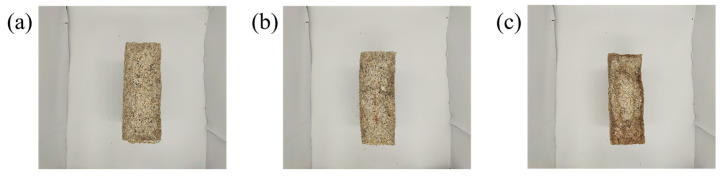
Sample images of Daqu at different grades. (**a**) Daqu-P; (**b**) Daqu-F; (**c**) Daqu-S.

The Daqu (QC) sample images were sourced from a light-flavor liquor factory. Considering the potential impacts of environmental conditions at different storage locations within the fermentation chamber, mature Daqu blocks were randomly sampled from the upper, middle, and lower layers of the storage room every day. This sampling method was carefully designed to ensure comprehensive coverage of different sample types throughout the entire sampling process. The Daqu samples were categorized into three grades: premium, first-grade, and second-grade [27,28]. To ensure balanced data distribution across different grades and maintain stability and robustness in modeling, 100 samples from each grade were selected for photography, resulting in a total of 300 Daqu images. Representative images of the three grades of QC samples are shown in Figure 1.

### 2.2. Image Acquisition Method

**Figure 2:**
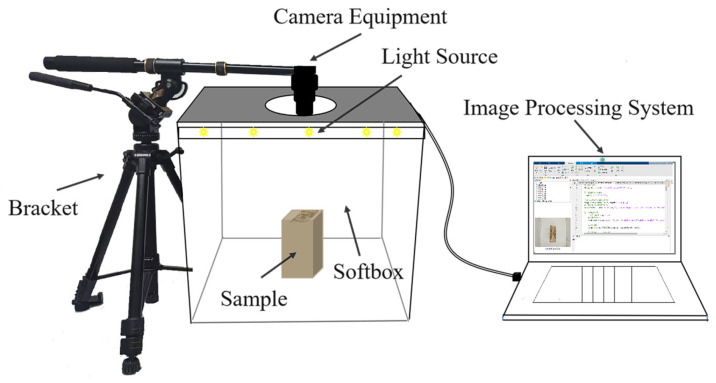
Computer vision system.

In order to better sample Daqu images and improve data accuracy, a low-cost image acquisition system based on an RGB camera and LED lights was developed. The system mainly consisted of a Complementary Metal-Oxide-Semiconductor (CMOS) camera (Canon (China) Co., Ltd., Beijing, China), support equipment, samples, two light sources (natural-light LEDs, 5500 K), a soft light box (400 mm × 400 mm × 400 mm), and an image processing platform. The system is shown in Figure 2. During image acquisition, the Daqu blocks were vertically sectioned, and the cross-section was placed directly beneath the camera to capture the cross-sectional features of each Daqu block as image analysis samples. To ensure the clarity and consistency of the images, the image acquisition environment was maintained under constant lighting conditions to avoid interference from external light sources [29]. All images were collected under the same system conditions and saved in JPG format, with a resolution of 6000 × 4000 pixels and an approximate image size of 4.5 MB.

### 2.3. Image Information Extraction

To construct classification models for Daqu-P, Daqu-F, and Daqu-S, image segmentation, region of interest (ROI) selection, and feature extraction methods were employed. These processes were conducted to process the data for Daqu classification and recognition, providing effective inputs for the classification models.

#### 2.3.1. Image Segmentation

**Figure 3:**
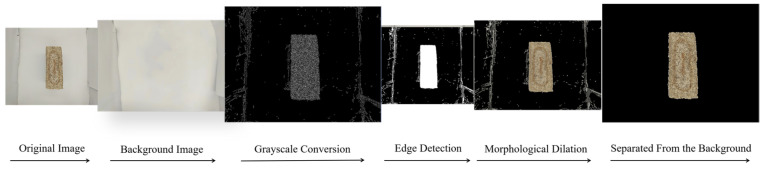
The steps of image segmentation.

Image segmentation divides the target image into multiple regions of interest or objects, performing preprocessing tasks such as resizing [30], background removal, grayscale conversion, and object separation, as shown in Figure 3 [31]. In this study, threshold segmentation, morphological fusion, and K-means clustering were selected as the three image segmentation methods.

In this study, a pixel intensity-based image segmentation method was used. The Otsu method [32] was employed, which determines an adaptive threshold by maximizing the inter-class variance between the foreground and background, effectively separating the target image from the background, thereby enhancing the accuracy and robustness of the feature extraction for Daqu images.

K-means clustering is an unsupervised machine learning algorithm. This method uses three initial data points as cluster centers and measures the Euclidean distance between the data points and the cluster centers to identify key image regions associated with Daqu grades, generating mask images to extract the target regions [33]. This processing approach helps identify the different color and texture features formed during the Daqu fermentation process.

The morphological fusion method, which combines the Canny edge detection algorithm [34,35] and morphological operations [36], was used to segment the target regions in different areas of the image. The study utilized edge detection to extract the target image’s edge information, applied morphological dilation to connect broken edges, filled holes, and removed regional noise, thereby obtaining more complete target regions and effectively reducing the interference of background noise in feature extraction [37].

#### 2.3.2. Selection of Regions of Interest

The region of interest (ROI) refers to a specific area selected for further analysis in image or video processing. By focusing on key regions of an image, ROI helps improve processing efficiency, enhance the accuracy of analysis, and reduce the influence of the background. The raw, starchy part of the surface of the Daqu block is called the “Pizhang”. During the fermentation process of Daqu, factors such as temperature, moisture, and time can cause differences in the central region and the thickness of the Pizhang, leading to the classification of Daqu into Daqu-P, Daqu-F, and second-grade categories based on these variations. A good fermentation environment promotes the growth and reproduction of microorganisms, causing the cross-section of the Daqu to appear grayish-blue without any discoloration and with or without a thin Pizhang, which is classified as Daqu-P. When excessive fermentation, high temperatures, or improper fermentation time cause the center of the cross-section to show a red spot or line, with a small dark area formed in the edge region and a thin Pizhang, it is classified as Daqu-F. If excessive moisture loss occurs, causing the Daqu to crack and leading to the growth of undesirable microorganisms, the cross-section will show cracks or black circles, and the dark areas at the edges will become more prominent with a larger Pizhang coverage area, which is classified as Daqu-S.

**Figure 4:**
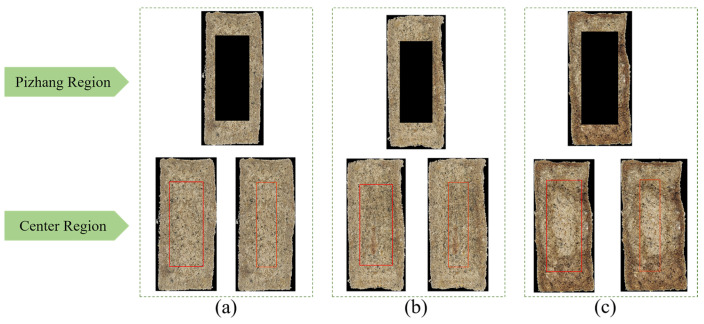
Visual representation of feature extraction from the ROI. (**a**) Daqu-P; (**b**) Daqu-F; (**c**) Daqu-S. The part circled by the red box indicates the center region.

Based on the previous analysis, the thickness of the Pizhang and the cross-sectional features were identified as key sensory criteria for evaluating the quality of Daqu. In this study, the Daqu images were divided into two ROIs based on the cross-sectional features and the Pizhang, representing the center region of the cross-section and the Pizhang region, respectively. Using an image binarization method, the boundaries of the Daqu sample area were detected, and the contours of the target image were identified to determine the minimum bounding rectangle, which was used to separate the ROIs from the acquired sample images. The feature distribution of the Daqu sample images was analyzed, and the rectangular area of interest was extracted based on the center coordinates of the target image. A width (200 pixels) × height (500 pixels) rectangle and a width (120 pixels) × height (500 pixels) rectangle were extracted as the center ROIs for the first layer classification and second layer classification tasks, with the remaining area representing the Pizhang ROI. Typical ROI images for different classification tasks are shown in Figure 4. The entire process aimed to accurately locate and analyze the key regions of the image, providing detailed feature information for subsequent image processing tasks.

#### 2.3.3. Image Feature Extraction

Feature extraction involves extracting representative features from target data for analysis, modeling, and prediction. During the production process of QC, the cross-section of Daqu-P typically appears grayish-blue, as shown in Figure 1a, which is one of the key indicators for distinguishing the quality of Daqu. During the drying stage, if the temperature exceeds the standard, it can lead to the growth of red mold, causing the center of the Daqu cross-section to appear red, as shown in Figure 1b. Evaporation of moisture can inhibit the normal growth of microorganisms, resulting in uneven fermentation, with black circles or cracks forming in the center of the Daqu and a dark area appearing at the edges, as shown in Figure 1c. Based on the color differences in Daqu of different grades, RGB and HSV color spaces were selected as the spatial features for the target images. The RGB features reflect the raw color information, while the HSV features capture variations in hue, saturation, and brightness, enabling more precise detection of Daqu’s color and texture characteristics under different conditions [38,39]. Therefore, the color space and pixel information of Daqu images were chosen to represent the different characteristics of Daqu images, as shown in Table 1.

### 2.4. Construction of Image Classification Models

In the experiments, due to slight differences in fermentation process control conditions, the Pizhang and cross-sectional features of Daqu-P and Daqu-F exhibited minimal variation. As a result, the performance of the model was suboptimal when directly applying a three-class classification approach. Therefore, a two-layer classification structure model for Daqu was proposed in this study.

#### 2.4.1. Feature Selection Methods

To reduce the dimensionality of image features, lower the computational complexity of algorithms, mitigate overfitting, and improve the model’s performance and computational efficiency, four feature selection methods were chosen for comparative study. These methods include the mean accuracy decrease method based on random forest (RF-MDA), recursive feature elimination (RFE), LASSO regression, and ridge regression.

The random forest mean decrease in accuracy (RF-MDA) is a commonly used feature importance measurement method that evaluates the contribution of each feature to the predictive performance of the random forest model by comparing the prediction accuracy with and without the feature [40].

Recursive feature elimination (RFE) works by recursively constructing models and gradually eliminating the least important features during each iteration, ultimately retaining the most significant feature subset [41]. In the classification of Daqu images, RFE can be used to progressively reduce the feature set and identify the most effective features for distinguishing different Daqu grades.

LASSO regression is a regularization method that achieves feature selection by introducing an L1 norm regularization term into the loss function [42,43]. The objective of LASSO is to minimize the following loss function:

(1) $$\mathrm{minimize}\left(\sum_{i=1}^{n}{\left(y_{i}-{\hat{y}}_{i}\right)}^{2}+\lambda \sum_{j=1}^{p}\left|\beta \left.j\right|\right.\right)\;$$

Ridge regression is similar to LASSO regression and is also a type of regularized regression; however, it introduces an L2 norm regularization term into the loss function [44]. Its objective function is the following:

(2) $$\mathrm{minimize}\left(\sum_{i=1}^{n}{\left(y_{i}-y_{i}\right)}^{2}+\lambda \sum_{j=1}^{p}\beta_{j}^{2}\right)$$

In this context, *y_i_* represents the actual values, *i* denotes the predicted values, *β_j_* are the model coefficients, and *λ* is the regularization parameter. In ridge regression, the regularization term *λ* controls the complexity of the model while reducing the adverse effects of multicollinearity on the estimation of model coefficients. In this study, *λ* was determined through a process of 10-fold cross-validation. A range of *λ* values from 0.01 to 100 with 100 logarithmically equally spaced points was defined. For each *λ* value in this range, a Gaussian process regression model with a squared exponential kernel was fitted to the data, and 10-fold cross-validation was performed. The mean squared error (MSE) was calculated for each *λ*, and the *λ* that minimized the MSE was selected as the optimal value. In the classification of Daqu images, ridge regression was employed to handle image features that were highly correlated.

#### 2.4.2. Model Construction Method

**Figure 5:**
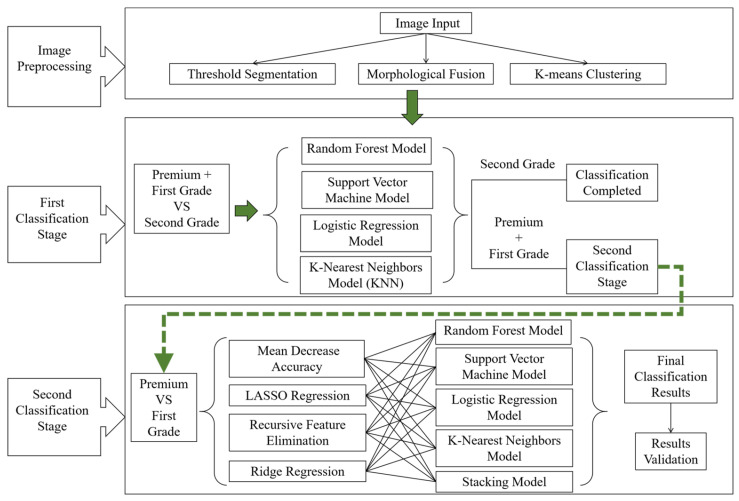
The flowchart of Daqu grade classification based on computer vision and machine learning.

In the first layer classification stage, Daqu-P and Daqu-F were combined into a single category and classified against Daqu-S. In the second layer classification stage, Daqu-P and Daqu-F were further differentiated. In the first layer classification stage, support vector machine (SVM), random forest (RF), logistic regression (LR), and k-nearest neighbors (KNN) algorithms were employed to construct the evaluation model for classifying Daqu-P, Daqu-F, and Daqu-S. In the second layer classification stage, based on the results of the first layer classification model, feature factors were screened using RF-MDA, RFE, LASSO regression, and ridge regression methods. Subsequently, SVM, RF, LR, KNN, and stacking models were utilized to construct the secondary evaluation model to distinguish Daqu-P from Daqu-F. The overall workflow is shown in Figure 5. A brief introduction to the above classification methods is provided below:

SVM achieves the classification of different classes of samples by mapping data into a higher-dimensional space using kernel functions (e.g., linear kernel, radial basis function), where it finds the optimal hyperplane that separates the data linearly. The objective is to maximize the margin between the hyperplane and the nearest support vectors in the feature space [45,46].

RF is an ensemble learning algorithm that constructs multiple decision trees by training on different subsets of the training data. Random feature selection is performed for each decision tree to reduce correlation among the trees. The final prediction is made by aggregating the results of individual decision trees, which enhances the model’s robustness and mitigates overfitting [47].

LR is a statistical model commonly used for binary classification problems. It predicts class membership by applying a linear combination of the input features to the sigmoid function, and the output probability is classified into different categories based on a set threshold [46,48].

KNN classifies new samples by calculating the distance between the new sample and the samples in the training set, selecting the K-nearest neighbors, and making a classification decision based on the majority vote of these neighbors [20,49].

Stacking is an effective ensemble learning technique that combines the predictions of multiple base learners to improve overall model performance [30,50]. In this study, the stacking model consisted of two layers: the base layer and the meta layer. In the base layer, RF, LR, and KNN were independently trained on the same training dataset and generated preliminary predictions. The predictions from these models were aggregated to form a new feature set, referred to as meta-features. The meta layer introduced RF and LR as meta-models, which were trained and used for prediction based on the meta-features generated from the base layer. Two stacking models were constructed and named S-RF and S-LR, respectively.

#### 2.4.3. Model Evaluation Metrics

The evaluation metrics selected for assessing the model’s reliability and stability include accuracy, precision, recall, F1 score, ROC curve, and AUC [51]. The calculations for these evaluation parameters are as follows:

(1) Accuracy measures the proportion of correctly classified data points by the model across all data.


(3) $$\mathrm{Accuracy}=\frac{\mathrm{TP}+\mathrm{TN}}{\mathrm{TP}+\mathrm{TN}+\mathrm{FP}+\mathrm{FN}}$$

(2) Precision measures how many of the predicted positive instances by the model are actually true positive instances.


(4) $$\mathrm{Precision}=\frac{\mathrm{TP}}{\mathrm{TP}+\mathrm{FP}}$$

(3) Recall measures how many of the actual positive instances are correctly classified as positive. It emphasizes the model’s ability to detect positive instances.


(5) $$\mathrm{Recall}=\frac{\mathrm{TP}}{\mathrm{TP}+\mathrm{FN}}$$

(4) F1 score is the harmonic mean of precision and recall, providing a balance between the two metrics (Equation (6)).


(6) $$F1=2\times \frac{Precision\times Recall}{Precision+Recall}$$

(5) The ROC curve (Receiver Operating Characteristic Curve) displays the performance of a model as the classification threshold varies by plotting the True Positive Rate (TPR) against the False Positive Rate (FPR).

True Positive Rate (TPR, equivalent to Recall):

(7) $$\mathrm{TPR}=\frac{\mathrm{TP}}{\mathrm{TP}+\mathrm{FN}}$$

False Positive Rate (FPR):

(8) $$FPR=\frac{FP}{FP+TN}$$

(6) AUC (Area Under the ROC Curve) is a numerical representation of the area under the ROC curve; a value closer to 1 indicates better model performance.


(9) $$\mathrm{AUC}=\int_{0}^{1}\mathrm{TPR}\left(x\right)d\left(\mathrm{FPR}\left(x\right)\right)$$

TP (True Positive): True positive class (correctly predicted positive class)

TN (True Negative): True negative class (correctly predicted negative class)

FP (False Positive): False positive class (incorrectly predicted as a positive class)

FN (False Negative): False negative class (incorrectly predicted as a negative class)

### 2.5. Environment Configuration and Data Partitioning

Environment configuration: The experiments were conducted on a system with a 12th Gen Intel(R) Core (TM) i5-1240P 1.70 GHz CPU, 16 GB RAM, running a 64-bit Windows 11 operating system. The computational environment used MATLAB 2010b software along with the Image Processing Toolbox and the Statistics and Machine Learning Toolbox.

To ensure the objectivity and reliability of model training, tuning, and evaluation results, stratified sampling was employed to split the dataset. The dataset was divided into training, validation, and testing sets with a ratio of 60%, 20%, and 20%, respectively. The training set was used for parameter learning and model training, the validation set was used for hyperparameter tuning and model performance assessment, and the test set was used for the final evaluation of the model’s generalization capability. When the model was evaluated, the cross-validation method was applied to the calculation of various evaluation metrics on the training set, the validation set, and the test set. The model was trained and evaluated through multiple different data partitions to ensure the reliability and stability of the evaluation results and avoid evaluation biases caused by a single data partition method.

## 3. Results and Analysis

### 3.1. Daqu RGB Color Distribution Features

**Figure 6:**
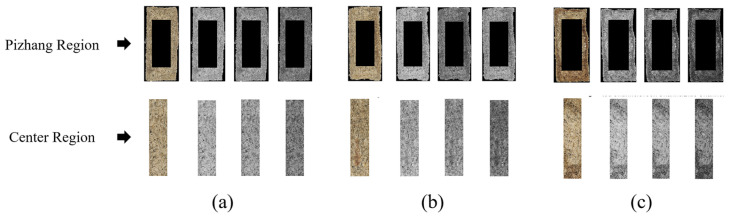
RGB distribution map. (**a**) Daqu-P; (**b**) Daqu-F; (**c**) Daqu-S. From left to right: RGB image, red channel histogram, green channel histogram, blue channel histogram.

**Figure 7:**
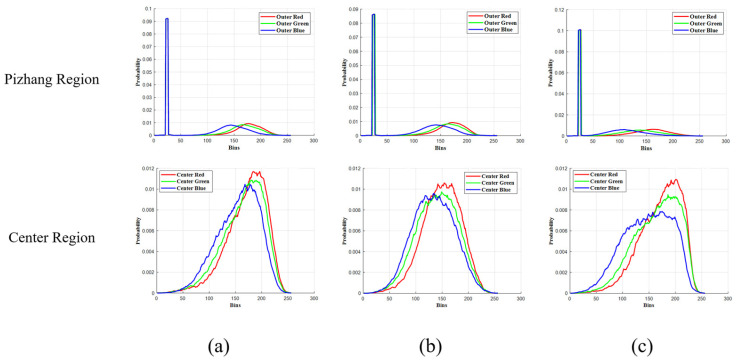
RGB histogram curves. (**a**) Daqu-P; (**b**) Daqu-F; (**c**) Daqu-S.

Figure 6 shows the image segmentation results of the Pizhang and center regions of Daqu samples at different grades. Figure 7 shows the RGB histogram curves of Daqu images at different grades, revealing the color differences between the Pizhang and center features. From the image segmentation results, it can be observed that the Pizhang and center region images of Daqu-P and Daqu-F display no significant difference in their distribution, while the image differences in the Daqu-S are more pronounced. Based on the histogram distribution analysis (Figure 7), the RGB histogram distributions of the Pizhang region of Daqu-P and Daqu-F are similar, with the central pixel points and distribution trends showing a close resemblance. However, the Pizhang region of Daqu-S demonstrates pixel accumulation that is uniformly distributed between intervals. The RGB histograms of the central regions of Daqu with different grades reveal significant differences. Daqu-P exhibits a sharp peak with a slight offset in the central pixel position, reflecting a relatively concentrated substance or characteristic in its composition or microstructure. From a flavor perspective, this concentration may be associated with the aggregation of certain key microbial communities that generate unique flavors, endowing Daqu-P with distinct flavor characteristics. In contrast, the peak of Daqu-F is flatter, and the difference in the central pixel position is minimal. This likely indicates that the microbial communities or chemical components within Daqu-F are relatively more balanced, lacking obvious dominant components or aggregation phenomena. In terms of quality, the uniform distribution of components generally implies that the fermentation process is more stable, and the quality is relatively easier to control and predict. Daqu-S shows an increasing peak and a wider pixel distribution range, indicating that its pixels accumulate relatively uniformly in different color intervals without distinct dominant color intervals. This may reflect that the components within Daqu-S are relatively complex, containing a variety of substances with different types and contents and without obvious dominant components. From a flavor perspective, this complex component distribution may result in Daqu-S having a rich flavor, with various flavors interwoven. Nevertheless, the flavor stability may be slightly compromised due to the complexity of the components. The differences in the cumulative distributions of pixel color intensities in the Pizhang and central regions of different grades provide effective visual evidence for the classification and grading of Daqu images.

### 3.2. The Impact of Image Segmentation Methods on Image Classification

**Figure 8:**
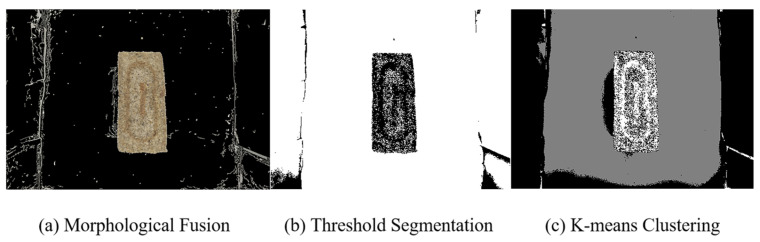
Image segmentation results.

The segmentation results of an image determine the quality of the image dataset, which in turn affects the final prediction performance of the model. Therefore, image segmentation is a crucial step in image classification. In this study, three different image segmentation methods—morphological fusion, threshold segmentation, and K-means clustering—were applied to process the images. The segmentation results of the three methods are shown in Figure 8.

Analysis of the segmentation results in Figure 8: Due to the presence of debris around the Daqu during image acquisition, the boundaries exhibit prominent husks and wheat straw. The morphological fusion method, through dilation to connect broken edges and fill holes, accurately extracted the boundaries and details of the Daqu image, filtering out noise and successfully separating the target image. As the research by Johnas Omanwa Maranga et al. [52] demonstrated, the effectiveness of the Canny edge detection algorithm in assisting image feature extraction was proven. The threshold segmentation method, by applying binarization, divides the image into the foreground and background, effectively extracting the Daqu outline. However, the internal details of the Daqu were ignored, resulting in a rough segmentation that is not suitable for images with complex textures. The K-means clustering segmentation method divides the image into multiple grayscale levels, but due to its sensitivity to lighting variations and shadows, the shadow areas were mistakenly recognized as the target, leading to inaccurate segmentation and blurred boundaries. In summary, the morphological fusion method is more suitable for Daqu image segmentation.

To compare the impact of different segmentation methods on model training results, the logistic regression algorithm was chosen as the classifier for analysis [53]. For each of the Daqu-P, Daqu-F, and Daqu-S images, 100 images were selected, with 14 feature factors extracted from each image. The two-grade classification sample weights were set to 1 and 2, resulting in 2800 data points for Daqu-P and Daqu-F and 2800 data points for Daqu-S. The model was evaluated in six aspects: accuracy, precision, recall, F1 score, AUC, and runtime, as shown in Table 2. The morphological fusion method achieved accuracy, precision, recall, and F1 scores of 96.67%, 95.00%, 95.00%, 0.95, and 0.96, respectively, improving by 8.62%, 14.79%, 10.53%, 12.63%, and 0.52%, respectively, over the threshold segmentation and by 44.83%, 57.38%, 10.53%, 40.10%, and 31.84%, respectively, over the K-means clustering methods. In terms of runtime, the morphological fusion method took longer than threshold segmentation but provided better real-time performance than K-means clustering. Considering the model evaluation metrics, the morphological fusion method provided the best segmentation results for Daqu images, successfully capturing the outlines and structural information of the images, offering clear boundaries, and accurately identifying the objects within the images.

### 3.3. Performance Evaluation of Classification Models

#### 3.3.1. Performance Evaluation of First-Layer Classification Models

**Figure 9:**
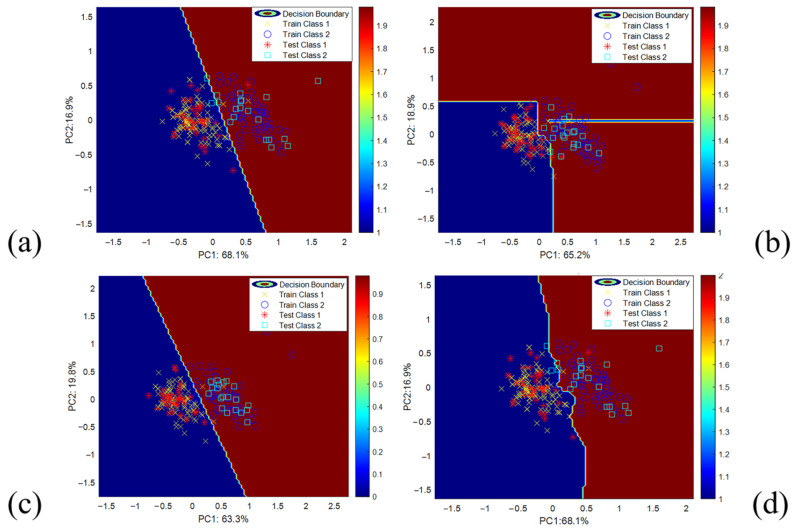
PCA low-dimensional data point distribution and model decision boundaries in the first layer classification of Daqu images: (**a**) SVM; (**b**) RF; (**c**) LR; (**d**) KNN.

To visually demonstrate the ability of the models to distinguish between samples, high-dimensional feature data were reduced to a two-dimensional space using Principal Component Analysis (PCA), allowing for the observation of data distribution in the low-dimensional space and the classification performance of the models. Based on the dimensionality-reduced feature data, Daqu-F classification models were constructed using SVM, RF, LR, and KNN, and decision boundaries were plotted using the model decision functions. The red and blue areas represent the classification regions for different grades in the first layer classification, as shown in Figure 9.

The decision boundaries of the SVM model and LR model exhibit a linear distribution, while the decision boundary of the RF model forms a “staircase-like” structure consisting of multiple straight lines. The decision boundary of the KNN model displays an irregular curved distribution. The decision boundaries of different models reflect their ability to classify Daqu samples of two different grades. The random forest and KNN models have more flexible decision boundaries, making them suitable for handling complex data distributions, while the SVM and LR models are more suitable for data with clearly defined linear distributions.

**Figure 10:**
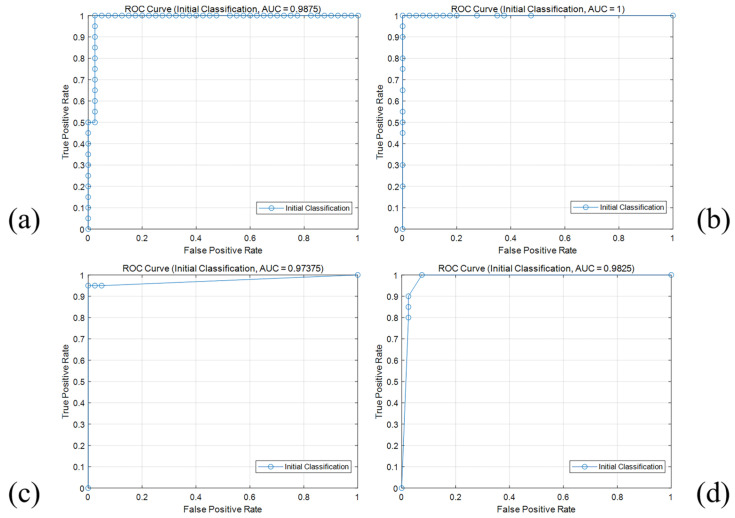
ROC curves for different models in the first layer classification. (**a**) SVM; (**b**) RF; (**c**) LR; (**d**) KNN.

Table 3 presents the classification results and model runtime of the SVM, RF, LR, and KNN models for Daqu image classification. The ROC curves for different models predicting Daqu grades are shown in Figure 10. The analysis results indicate that the SVM and RF models have identical accuracy, precision, recall, and F1 scores, meaning that they can accurately identify most positive samples while maintaining a low false positive rate, demonstrating excellent discrimination ability. However, the AUC of the RF model is 1.01% higher than that of the SVM, which corresponds to the ROC curve results. The non-linear decision boundary of the RF model and the high-dimensional projection ability of the SVM make them more suitable for capturing the complex color and regional differences in Daqu images. The LR model has the same accuracy as the previous two models, but its precision, recall, and F1 scores are slightly lower, reflecting the linear model’s slightly poorer adaptability to complex distributions and its weaker ability to adjust to color distribution changes in Daqu images. The KNN model performs slightly worse in terms of precision compared to the other models, possibly due to its sensitivity to data noise. Overall, the RF model performs excellently across all metrics, and the PCA dimensionality reduction results are consistent with the model evaluation outcomes. The random forest model exhibits stronger adaptability in the reduced-dimensional space and can effectively capture and distinguish subtle color differences and regional features in Daqu images.

#### 3.3.2. Feature Selection Results for the Second Layer Classification

To enhance the classification between Daqu-P and Daqu-F, a total of 200 images were selected (100 images each for Daqu-P and Daqu-F). For each image, 38 feature factors were extracted according to Table 2, resulting in 3800 data points for each grade. The selection of key features directly influenced the model’s performance. In this study, feature selection was performed using four methods: random forest, recursive feature elimination (RFE), LASSO regression, and ridge regression. The selected features were numbered in the order presented in Table 4.

Table 4 presents the importance scores of features selected by different methods. It can be observed that various feature selection methods emphasize different aspects of the center region and Pizhang area of Daqu. The RF-MDA method assigned higher scores to features from both the central and Pizhang regions, with scores ranging narrowly between 0.41 and 0.59. High importance was noted for features numbered 1, 2, 11, 17, and 21, as well as 28, 34, and 38, indicating strong differentiation capability and suitability for identifying key features related to distinct grade differences in Daqu. The feature selection of RFE and ridge regression was more balanced, with central region features mainly focused on numbers 2, 4, 12, 17, and 24, while the Pizhang region focused on features numbered 27, aiding in the identification of critical distinguishing characteristics in Daqu. In contrast, LASSO regression exhibited lower scores with a smaller range, resulting in fewer selected features. Overall, RF-MDA demonstrated superior performance in identifying color and texture features associated with the fermentation process of Daqu, while RFE, ridge regression, and LASSO regression provided different choices based on feature quantity and importance scores. The feature importance scores reflect the complexity of the feature selection process and contribute to a better understanding of the different selection strategies employed by each method.

#### 3.3.3. Performance Evaluation of the Second Stage Classification Model

The selected feature factors from Section 3.3.2 were used to construct the dataset for the second-layer classification of Daqu. The dataset was split into training, validation, and test sets with a ratio of 60%, 20%, and 20%, respectively. Classification models, including SVM, RF, LR, KNN, S-RF, and S-LR, were then developed using different feature selection methods. The accuracy results for various combinations of feature factors and models are presented in Table 5. The RF model exhibited outstanding overall performance, particularly when RF-MDA and ridge regression were used as feature selection methods, achieving an accuracy of 87.50%. This result indicated the stability of the RF model in handling different feature combinations. The stacking models (S-LR and S-RF) effectively integrated the features of the Daqu center and Pizhang regions by leveraging the predictions of multiple models. When combined with the RF-MDA method, the S-RF model achieved an accuracy of 90.00%, demonstrating the significant contribution of the color features from the dark areas of the Daqu center and the texture features from the Pizhang regions. In contrast, the SVM model was more sensitive to feature selection methods, performing best with the RFE method, where it achieved an accuracy of 82.50%. This could be attributed to the RFE method’s ability to effectively select feature combinations suitable for SVM, emphasizing the most discriminative features of the Daqu center. However, under RF-MDA, LASSO, and ridge regression, the SVM model showed poorer adaptability to the selected features, with accuracy ranging between 70.00% and 80.00%. This further suggested that SVM is more suited for selecting specific key features but has limited adaptability to feature sets with high diversity. The LR model achieved an accuracy of 85.00% when combined with LASSO and ridge regression, reflecting the effectiveness of regularization methods in linear models. These feature selection methods ensured a parsimonious model while retaining critical information from the center and Pizhang regions. The KNN model showed relatively stable performance, achieving an accuracy of 80.00% when combined with LASSO and ridge regression. This result indicated that KNN had a higher adaptability to features with a uniform distribution of importance weights across different regions. Overall, the color distribution and structure of the Daqu center region and the color distribution of the Pizhang region contributed significantly to the improvement of model performance. The combination of RF-MDA and the S-RF model achieved the best performance. Furthermore, during the second layer classification stage, the S-RF model had a runtime of 2.49 s, which was moderate among all models. Compared to the LR model’s runtime of 4.12 s, the S-RF model required less time, highlighting its efficiency advantage.

**Figure 11:**
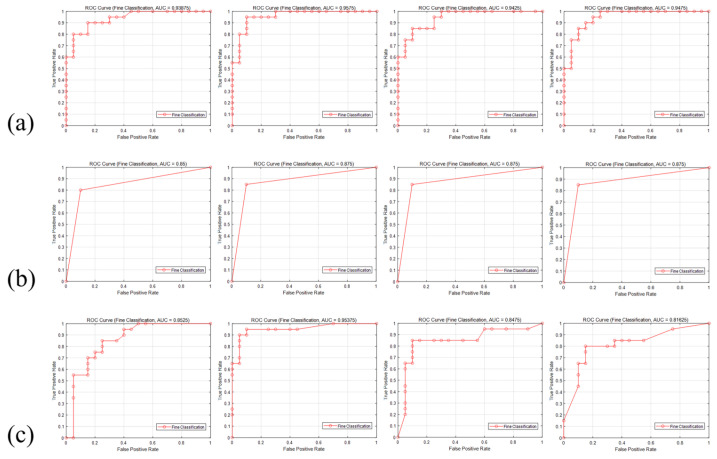
ROC curve (**a**) RF; (**b**) S-LR; (**c**) S-RF. The corresponding feature selection methods for the images are arranged in the following order: RFE, RF-MDA, LASSO regression, and ridge regression.

To comprehensively evaluate the effectiveness of various combinations, those with accuracy exceeding 85.00% were selected for further assessment using additional model evaluation metrics, as shown in Table 6. From the analysis of model performance, the S-RF model achieved the best results when features were selected using the RF-MDA method, with precision and F1 score improvements of 5.26% and 2.24%, respectively, over the S-LR and RF models. This indicates that the S-RF model exhibits higher stability and reliability in distinguishing between positive and negative samples. For the RFE method, the S-LR model demonstrated the highest precision and F1 score, outperforming the RF model and S-RF model by 5.26% and 2.38% and by 13.07% and 3.57%, respectively. In terms of recall and F1 score, the S-LR model also surpassed the other two models, achieving an F1 score of 0.84. The precision and AUC of the RF model decreased to 84.21% and 0.93875, suggesting that the RFE method may have excluded important features for classification. The S-RF model performed the worst under the RFE method, indicating the limited suitability of recursive feature elimination for ensemble models. Under the LASSO regression method, the performance of the S-RF and S-LR models was similar; however, the S-LR model achieved a 3.44% higher AUC, suggesting that LASSO regression effectively selected features beneficial for both linear and non-linear models. With ridge regression, the S-LR model performed the best, achieving a precision of 94.12%, which was 4.94% and 10.52% higher than the RF model and S-RF model, respectively. Other evaluation metrics for the S-LR model also demonstrated strong robustness. Among the four optimal combinations, the S-RF model paired with the RF-MDA method achieved the highest accuracy and AUC, at 94.44% and 0.95, respectively. The ROC curve of the models is shown in Figure 11. From the shape of the ROC curve, the combination of RF-MDA and S-RF displayed a larger area under the curve, indicating excellent classification performance, with the model effectively distinguishing between positive and negative samples. The combination of RF-MDA and the S-RF model performed best, as it was able to better capture effective features in the data, thereby enhancing classification performance.

### 3.4. Validation of the Model with Multiple Types of Daqu

To verify the applicability and generalizability of the model framework, images of HH and HX were classified. A total of 900 images were used, consisting of 150 samples each of premium, first-grade, and second-grade HH and HX. The same feature extraction methods and two-layer classification framework used for QC were applied. A comparative analysis of the classification performance for different types of Daqu was conducted, and the results are shown in Table 7.

Table 7 shows that in the first classification layer stage, the RF model achieved classification accuracies of 92.78% and 90.00% for HH and HX, respectively. The best-performing model for HH was KNN, with an accuracy of 95.56%, while the best-performing model for HX was SVM, with an accuracy of 92.22%. In the second layer classification stage, the S-RF model, combined with the RF-MDA method, achieved classification accuracies of 81.67% and 75.00% for HH and HX, respectively. The highest classification accuracy for HH was obtained using RF and LR models with the LASSO regression method, reaching 86.67%. For HX, the RF model combined with the ridge regression method achieved an accuracy of 85.00%, indicating that model performance for specific Qu types can be improved by adjusting models and factor selection methods. Compared to the classification model for QC, the classification accuracy for HH and HX was lower. This may be due to the more complex image features of HX and HH compared to QC. Specifically, the cross-sections of premium HX and HH often exhibit red regions or two brownish-black stripe features, while first-grade Qu cross-sections show varying degrees of cracks, smaller red core areas, or less distinct two brownish-black stripe features. These complex and similar image characteristics made it more challenging for the model to accurately classify sample categories. Additionally, due to visual differences in the image features of different Daqu types, the models selected different feature factors during training, resulting in optimal models for HH and HX differing from those for QC. The classification results of HH and HX demonstrate that the proposed model framework exhibits good applicability. As previous studies demonstrated, machine learning algorithms are applicable to the quality grading of various agricultural products [21]. It can assist the brewing industry in controlling the quality of Daqu and improving production efficiency and product stability. However, in practical applications, the implementation of the model faces challenges in data collection and modification as well as the interpretability of the model. Subsequent research work can utilize intelligent data collection devices and visualization technologies to enhance the adaptability of the model.

## 4. Conclusions

In this study, a two-layer classification strategy for Daqu based on computer vision and machine learning algorithms was proposed. This strategy effectively reduced the classification difficulty and significantly improved classification accuracy, demonstrating the advantages of hierarchical classification in addressing complex grade classification tasks. Based on the acquisition of Daqu-P, Daqu-F, and Daqu-S images as regions of interest, three methods—morphological fusion, threshold segmentation, and K-means clustering—were compared. The morphological fusion method was determined to be the most suitable segmentation method for Daqu classification. Four feature selection methods, including RF-MDA, RFE, LASSO regression, and ridge regression, were applied to optimize feature extraction for the model. The classification performance of five models, namely SVM, RF, LR, KNN, and stacking models, was evaluated. In the first layer classification, the random forest model showed superior performance in classifying Daqu samples, with an accuracy, precision, recall, F1 score, and AUC of 96.67%, 97.50%, 97.50%, 0.97, and 0.99, respectively. The accuracy was 1.72% higher than that of KNN, while the precision and recall were both 2.56% higher than those of LR. In the second layer classification, the combination of RF-MDA and S-RF achieved the best results across all evaluation metrics, with an accuracy and precision of 90.00% and 94.44%, respectively. The models that performed well in both classification stages included the random forest model, which not only excelled in the first layer but also played a key role in feature selection. The stacking model, by combining the results of multiple base models—RF, LR, and KNN—leveraged their strengths to further enhance performance. Notably, when random forest was used as the meta-model, all performance metrics of the stacking model reached optimal values. This indicates that RF is not only effective as a standalone model but can also be further optimized through stacking frameworks. Finally, through the training and validation of models for different types of Daqu, specifically HH and HX, the results indicated that the grading and classification processes, as well as the models, demonstrated good applicability. These findings indicate that applying computer vision and machine learning to Daqu grade prediction is both feasible and effective. Future research could further explore joint modeling for a wider variety of Daqu types, enhancing the model’s performance in practical production environments and providing technical support for quality control in the liquor-making industry.

## Figures and Tables

**Table 1:** Feature factor statistics.

Classification Steps	Factor Type	Factor Name	Quantity (Count)	Total (Count)
First layer classification	RGB	MeanRed, MeanGreen, MeanBlue, StdRed, StdGreen, StdBlue	6	14
	HSV	MeanHue, MeanSaturation, MeanValue, StdHue, StdSaturation, StdValue	6	
	Pixel	RectArea, DarkArea	2	
Second layer classification	RGB	MeanRed, MeanGreen, MeanBlue, StdRed, StdGreen, StdBlue	6	38
	HSV	MeanHue, MeanSaturation, MeanValue, StdHue, StdSaturation, StdValue	6	
	Pixel	RectArea, DarkArea	2	
	RGB Color Histogram (2 ROIs)	Red_Bin1, Red_Bin2, Red_Bin3, Red_Bin4, Green_Bin1, Green_Bin2, Green_Bin3, Green_Bin4, Blue_Bin1, Blue_Bin2, Blue_Bin3, Blue_Bin4	24	

Note: The RGB color histogram divides the pixel value range (0–255) for each channel into four bins (bin1: 0–63, bin2: 64–127, bin3: 128–191, bin4: 192–255).

**Table 2:** Model evaluation of image segmentation methods.

Method	Accuracy (%)	Precision (%)	Recall (%)	F1 Score	AUC	Image Processing Time (s)
Threshold Segmentation	88.33	80.95	85.00	0.83	0.96	103.29
K-means Clustering	53.33	40.48	85.00	0.55	0.65	401.11
Morphological Fusion	96.67	95.00	95.00	0.95	0.96	130.78

**Table 3:** Evaluation metrics for the first layer classification model.

Model	Accuracy (%)	Precision (%)	Recall (%)	F1 Score	AUC	Runtime (s)
SVM	96.67	97.50	97.50	0.97	0.98	0.15
RF	96.67	97.50	97.50	0.97	0.99	3.80
LR	96.67	95.00	95.00	0.95	0.96	0.33
KNN	95.00	95.12	97.50	0.96	0.98	0.45

**Table 4:** Feature importance of different feature selection methods.

Feature Area	RFE		LASSO Regression		RF-MDA		Ridge Regression	
	Factor Number	Importance Score	Factor Number	Importance Score	Factor Number	Importance Score	Factor Number	Importance Score
Center	1	-	1	0.0019	1	0.5901	1	0.2025
	2	-	2	-	2	0.5499	2	0.3675
	3	-	3	-	3	0.4303	3	0.1097
	4	0.3575	4	0.0224	4	0.4270	4	-
	5	-	5	0.2149	5	0.4723	5	-
	6	-	6	-	6	-	6	0.2165
	7	0.2117	7	0.1480	7	-	7	0.1875
	8	-	8	0.2509	8	1.0250	8	0.3576
	9	-	9	0.2171	9		9	0.1930
	10	-	10	0.0035	10	0.4881	10	0.2027
	11	0.0845	11		11	0.5443	11	-
	12	0.1368	12	0.2075	12	0.4144	12	0.3603
	13	-	13	0.1189	13	-	13	0.1080
	15	-	15	-	15	-	15	-
	16	0.2285	16	-	16	-	16	0.1120
	17	0.2919	17	0.1105	17	0.5430	17	0.4287
	18	0.1256	18	-	18	-	18	-
	19	-	19	-	19	-	19	-
	20	0.1624	20	-	20	-	20	0.1395
	21	0.0389	21	-	21	0.5716	21	-
	22	0.1227	22	-	22	0.5321	22	0.1637
	23	-	23	-	23	-	23	-
	24	0.3772	24	0.1008	24	0.5370	24	0.2610
	25	0.3365	25	0.0642	25	-	25	0.2377
	26	0.3089	26		26	-	26	0.1806
Pizhang	14	-	14	0.3452	14	-	14	-
	27	0.4121	27	0.3395	27	-	27	0.4351
	28	-	28	0.0670	28	0.4175	28	0.1119
	29	0.1381	29	-	29	-	29	-
	30	-	30	0.1281	30	-	30	-
	31	-	31	-	31	-	31	-
	32	0.0201	32	-	32	-	32	-
	33	-	33	0.0253	33	-	33	-
	34	-	34	-	34	0.4150	34	-
	35	0.0116	35	0.0488	35	-	35	-
	36	0.0178	36	0.0443	36	-	36	-
	37	0.0526	37	0.0336	37	-	37	-
	38	0.0163	38	-	38	0.4148	38	
Total	20		20		16		19	

**Table 5:** Comparison of model accuracy in the second layer of classification.

Model	RF-MDA (%)	RFE (%)	LASSO Regression (%)	Ridge Regression (%)	Runtime (s)
SVM	70.00	82.50	77.50	80.00	0.09
RF	87.50	82.50	85.00	87.50	1.28
LR	72.50	80.00	85.00	85.00	4.12
KNN	70.00	77.50	80.00	80.00	0.29
S-LR	87.50	85.00	87.50	87.50	2.83
S-RF	90.00	80.00	87.50	82.50	2.49

**Table 6:** Results of different selection methods and model combinations in the second layer classification.

Method	Model	Precision (%)	Recall (%)	F1 Score	AUC
RF-MDA	RF	89.47	85.00	0.87	0.95
	S-LR	89.47	85.00	0.87	0.87
	S-RF	94.44	85.00	0.89	0.95
RFE	RF	84.21	80.00	0.82	0.93
	S-LR	88.89	80.00	0.84	0.85
	S-RF	77.27	85.00	0.81	0.85
LASSO Regression	RF	85.00	85.00	0.85	0.94
	S-LR	89.47	85.00	0.87	0.87
	S-RF	89.47	85.00	0.87	0.84
Ridge Regression	RF	89.47	85.00	0.87	0.94
	S-LR	94.12	80.00	0.86	0.87
	S-RF	84.21	80.00	0.82	0.81

**Table 7:** Classification accuracy of the model for different types of Daqu.

Model	First Layer Classification Stage	Second Layer Classification Stage			
		Feature Selection Methods			
		RF-MDA(%)	RFE(%)	LASSO Regression (%)	Ridge Regression (%)
(a) HH					
SVM	91.75	85.00	82.50	83.33	75.00
RF	92.78	68.00	80.00	86.67	76.67
LR	93.81	83.33	81.67	86.67	80.00
KNN	95.56	76.67	71.67	75.00	76.67
S-LR	-	78.33	73.33	76.67	70.00
S-RF	-	81.67	78.33	80.00	77.78
(b) HX					
SVM	92.22	68.00	70.00	68.00	63.00
RF	90.00	83.33	80.00	81.67	85.00
LR	86.00	83.33	83.33	81.00	81.67
KNN	86.00	70.00	68.00	69.00	75.00
S-LR	-	75.00	76.00	72.00	75.00
S-RF	-	75.00	76.00	73.00	74.00

## Data Availability

The data presented in this study are available on request from the corresponding author, however, the release of the data is restricted because it involves enterprise-internal data.
